# Loss of *atrx* cooperates with p53-deficiency to promote the development of sarcomas and other malignancies

**DOI:** 10.1371/journal.pgen.1008039

**Published:** 2019-04-10

**Authors:** Felix Oppel, Ting Tao, Hui Shi, Kenneth N. Ross, Mark W. Zimmerman, Shuning He, Guangxiang Tong, Jon C. Aster, A. Thomas Look

**Affiliations:** 1 Department of Pediatric Oncology, Dana-Farber Cancer Institute, Harvard Medical School, Boston, MA, United States of America; 2 Heilongjiang River Fisheries Research Institute of Chinese Academy of Fishery Sciences, Harbin, China; 3 Department of Pathology, Brigham and Women's Hospital, Harvard Medical School, Boston, MA, United States of America; UNITED STATES

## Abstract

The SWI/SNF-family chromatin remodeling protein ATRX is a tumor suppressor in sarcomas, gliomas and other malignancies. Its loss of function facilitates the alternative lengthening of telomeres (ALT) pathway in tumor cells, while it also affects Polycomb repressive complex 2 (PRC2) silencing of its target genes. To further define the role of inactivating *ATRX* mutations in carcinogenesis, we knocked out *atrx* in our previously reported *p53/nf1*-deficient zebrafish line that develops malignant peripheral nerve sheath tumors and gliomas. Complete inactivation of *atrx* using CRISPR/Cas9 was lethal in developing fish and resulted in an alpha-thalassemia-like phenotype including reduced alpha-globin expression. In *p53/nf1*-deficient zebrafish neither peripheral nerve sheath tumors nor gliomas showed accelerated onset in *atrx*+/- fish, but these fish developed various tumors that were not observed in their *atrx*+/+ siblings, including epithelioid sarcoma, angiosarcoma, undifferentiated pleomorphic sarcoma and rare types of carcinoma. These cancer types are included in the AACR Genie database of human tumors associated with mutant *ATRX*, indicating that our zebrafish model reliably mimics a role for *ATRX*-loss in the early pathogenesis of these human cancer types. RNA-seq of *p53/nf1-* and *p53/nf1/atrx-*deficient tumors revealed that down-regulation of telomerase accompanied ALT-mediated lengthening of the telomeres in *atrx*-mutant samples. Moreover, inactivating mutations in *atrx* disturbed PRC2-target gene silencing, indicating a connection between ATRX loss and PRC2 dysfunction in cancer development.

## Author summary

Somatic mutations in genes coding for epigenetic regulators such as *ATRX* are found across a diverse group of cancer types, suggesting their broad relevance in tumor induction and progression. However, tumors that have been linked to these chromatin remodelers can arise in many different molecular and cellular contexts, requiring studies with new experimental models to understand the extent and mechanisms of tumor development mediated by these regulatory proteins. Thus, we analyzed the tumor suppressive role of *atrx* in zebrafish that already harbored inactivating mutations of *p53* and *nf1*. Homozygous deletion of *atrx* was lethal in developing fish, whereas the partial loss of this gene (*atrx+/-*) within the *p53/nf1-*deficient background led to a diverse spectrum of tumors not observed in animals that were wildtype for *atrx*, including epithelioid sarcoma, angiosarcoma, and rare carcinomas. Most of the cancer types we identified correspond to human tumors in the *ATRX*-mutant tumor sample cohort within the AACR Genie database, attesting to the relevance of our findings to human cancer. Further analysis revealed downregulation of telomerase during the lengthening of the telomeres through the ALT pathway, and disturbed function of the polycomb repressive complex 2 as key mechanistic components underlying *atrx*-linked tumorigenesis. These results demonstrate how a *p53/nf1* compromised genetic background combined with *ATRX* haploinsufficiency leads to a broad spectrum of sarcomas and carcinomas associated with loss of this chromatin modulator.

## Introduction

The alpha thalassemia/mental retardation syndrome X-linked (ATRX) protein is involved in the epigenetic regulation of gene expression. It is classified as a SWI/SNF-family chromatin remodeling factor due to its ATP-dependent helicase domain. In humans, germline loss of *ATRX* function causes mental retardation and alpha thalassemia that is associated with reduced alpha globin expression levels, lower blood-oxygen levels and hypochromia, anisocytosis, and poikilocytosis of red blood cells [1–5]. Because the *ATRX* gene is located on the X-chromosome in humans, females can carry a mutant allele heterozygously, without developing symptoms. Loss of *ATRX* leads to reduced levels of histone 3.3 (H3.3) incorporation, telomere destabilization and increased homologous recombination facilitating the development of ALT. ATRX binds to the death domain-associated protein 6 (DAXX) and recognizes H3K9me3 marks with its cysteine-rich domain termed ADD (ATRX-DNMT3-DNMT3L) [6]. The ATRX/DAXX-mediated deposition of H3.3 maintains the condensed heterochromatic state [7,8].

ATRX also guides the Polycomb repressive complex 2 (PRC2) to its targets for gene silencing by tri-methylation of histone 3 lysine 27 (H3K27me3) [9], a repressive epigenetic mark established by PRC2 [10]. This process is crucial for X-chromosome inactivation, mediated by the noncoding RNA *XIST* [11], which is expressed only from the to-be-silenced X-chromosome (Xi) and spreads along the Xi in *cis* [12]. ATRX binding to *XIST* is essential for PRC2 recruitment following *XIST* in *cis* along the Xi and also functions as an adaptor that affects PRC2 function beyond Xi inactivation. Upon *ATRX* knockdown in a human fibroblast cell line, PRC2 is unable to silence its target genes by deposition of the H3K27me3 mark at specific sites within the gene body. Instead, H3K27me3 is established at ectopic sites in the intergenic space and at non-canonical sites in the target genes, demonstrating the importance of ATRX for normal gene silencing by PRC2 [9].

Over the past 5 years, it has become apparent that mutations in epigenetic regulator genes are involved in the onset and progression of a large number of malignancies. The loss of *ATRX* in gliomas [13], neuroendocrine tumors [14] and various sarcoma types [15–19] facilitates alternative lengthening of the telomeres (ALT) and thereby stabilizes the genome of cancer cells during cancer development, a crucial step in the immortalization of cancer cells in general and a requirement for the formation of malignant tumors in humans [20]. There are two mutually exclusive mechanisms to elongate telomeres in tumor cells, i) telomerase (TERT) re-expression and ii) ALT activation. Individual tumor types differ in the frequency with which these mechanisms are activated [21]. For example, gliomas more frequently re-express TERT, but about 80% of ALT-positive pediatric high-grade gliomas are *ATRX*-deficient [13]. Comparable results demonstrating a strong association between *ATRX* deficiency and ALT were obtained in pancreatic neuroendocrine tumors (PanNETs) [22] and sarcomas [15–18]. ALT-positive cancer cell lines were recently found to preferentially have *ATRX* inactivation [23,24].

The mechanisms underlying ALT activation upon *ATRX* loss are not fully understood. It has been proposed that the heterochromatic state of the telomeres is disrupted when H3.3 can no longer be loaded by ATRX/DAXX, so that chromatin opens up and telomeres become more accessible [25,26]. Others have shown that ALT depends on telomere elongation by homologous recombination-mediated DNA replication (HR) [27]. It was has also been shown in a genetic mouse model of glioma that *ATRX* deficiency results in reduced activity of the non-homologous end joining pathway, which competes with HR in DNA repair [28]. This suggests that there may be increased HR-activity in ATRX-depleted cells, which further facilitates ALT.

Loss-of-function (lof) mutations in *ATRX* clearly promote the activity of ALT, which is an important step in establishing cellular immortality. However, the changes in tumor biology (other than activation of ALT) caused by *ATRX* loss are poorly understood. As ATRX loss is found in a variety of cancer types, we aimed to assess ATRX loss in combination with dysregulation of two of the most prevalent types of oncogenic pathways—the Ras pathway and the p53 pathway—to include a large spectrum of human cancers. Thus, we knocked out *ATRX* in the germline of the previously published *p53*- and *nf1*-deficient zebrafish model, in which mutants develop malignant peripheral nerve sheath tumors (MPNSTs) and high-grade gliomas [29]. Here we demonstrate that homozygous in activation of *atrx* leads to lethal alpha-thalassemia in zebrafish, while heterozygous loss of *atrx* in the context of combined *p53/nf1-*deficiency induces the onset of multiple histologic tumor types that are otherwise not observed in *nf1/p53*–deficient fish.

## Results and discussion

### CRISPR/Cas9-mediated knockout of *atrx*

**Fig 1:**
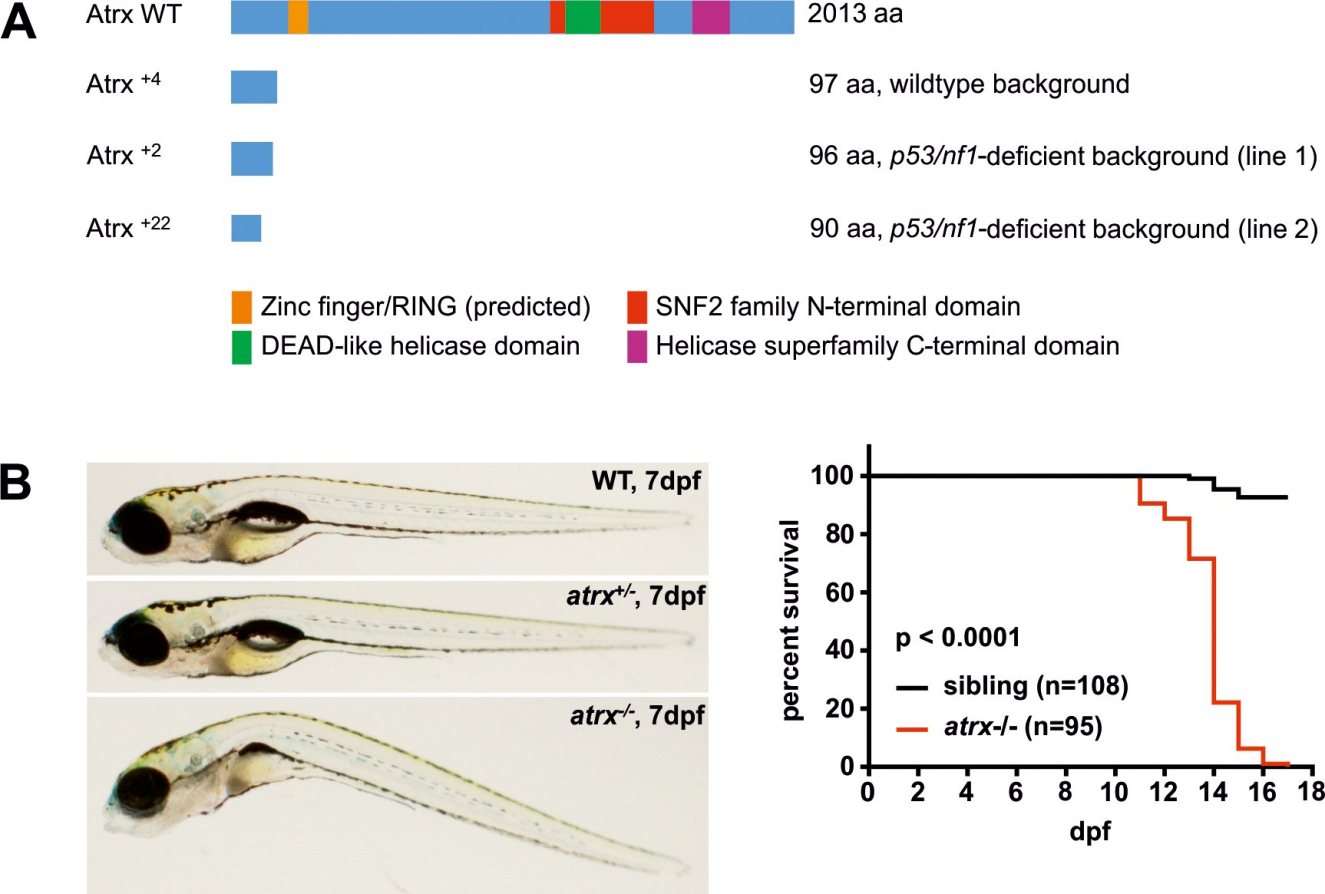
CRISPR/Cas9-mediated knockout of *atrx* in zebrafish germline. **(A)** The wildtype (WT) Atrx protein consists of 2013 amino acids (aa). Targeting of the *atrx* coding sequence with CRISPR/Cas9 resulted in truncation of Atrx before its functional domains and confers a loss of function; numbers indicate the underlying genetic alterations, e.g., +4 = insertion of 4 bases. **(B)** *atrx-/-* embryos show developmental abnormalities and die within 18 days post fertilization (dpf); sibling includes both *atrx+/-* and *atrx+/+* embryos.

To create loss-of-function (lof) mutations in *atrx* in zebrafish germline, we induced frameshift-mutations in exon 4 of *atrx* using CRISPR/Cas9 (S1 Fig and S1 Table). This resulted in a truncated Atrx protein lacking both ADD and ATPase/helicase domains (Fig 1A) which represents a total loss of Atrx function. Mutant alleles were generated in both wildtype (strain AB) zebrafish and in the previously published *nf1/p53*-deficient zebrafish line that expresses the green fluorescent protein (GFP) marker under the control of the zebrafish *sox10* promoter (*sox10*:*GFP*) [29].

After injection of gRNAs and *Cas9* mRNA into one-cell-embryos, fish were raised to fertility and out-crossed with non-injected siblings. All 10 analyzed injected F0 fish of the *p53/nf1*-deficient line transmitted mutations into the F1 generation. When 25 F1-embryos derived from these F0 fish were examined for their genomic alterations at the target locus, we observed a frameshift-mutation rate of 40%, with 30% harboring deletions and 70% harboring insertions (S1 Table). Moreover, 48% of zebrafish exhibited the in-frame deletion of a specific triplet, two had other in-frame mutations (8%) and the single remaining fish (4%) had a 3 bp substitution. The most recurrent in-frame mutation deleted codon 98 residing 4–6 bases upstream of the PAM-sequence. It is known that certain gRNAs recurrently induce specific genomic alterations in knockout-approaches using CRISPR/Cas9. Thus, genome editing was highly efficient and the resulting genomic alterations are consistent with previous studies [30,31]. We chose two zebrafish lines with frameshift mutations in the *p53/nf1*-deficient background and one line in the wildtype background to conduct all experiments described in this manuscript (S1 Fig and S1 Table). All our here presented knockout lines carry frameshift mutations in *atrx* in germline and thus model a total loss of ATRX, as it is frequently observed in human tumors [13–19,32]. As ATRX is a tumor suppressor, our knockout recapitulates most closely the situation in broad carcinogenesis. To our knowledge, specific point mutations disturbing certain functions of ATRX or gain-of-function mutations are not described so far.

F1 fish of all three lines reached fertility at around three months of age, but never produced viable adult offspring with the *atrx*-/- genotype. Genotyping of developing embryos revealed that *atrx*-/- embryos of all lines died between 10 and 18 days post fertilization (dpf) (Fig 1B). Each of these embryos displayed a body curvature phenotype and a lack of the swim bladder. The embryonic lethality observed with the *atrx*-/- genotype is also observed in mice [33–35]. In zebrafish, this is also true for *nf1* which is duplicated in fish, so that at least 1 out of 4 alleles of the two *nf1*-genes (either *nf1a* or *nf1b*) has to remain wildtype to enable normal development of the embryo [29].

### Knockout of *atrx* reduces globin expression and affects definitive erythropoiesis in zebrafish

**Fig 2:**
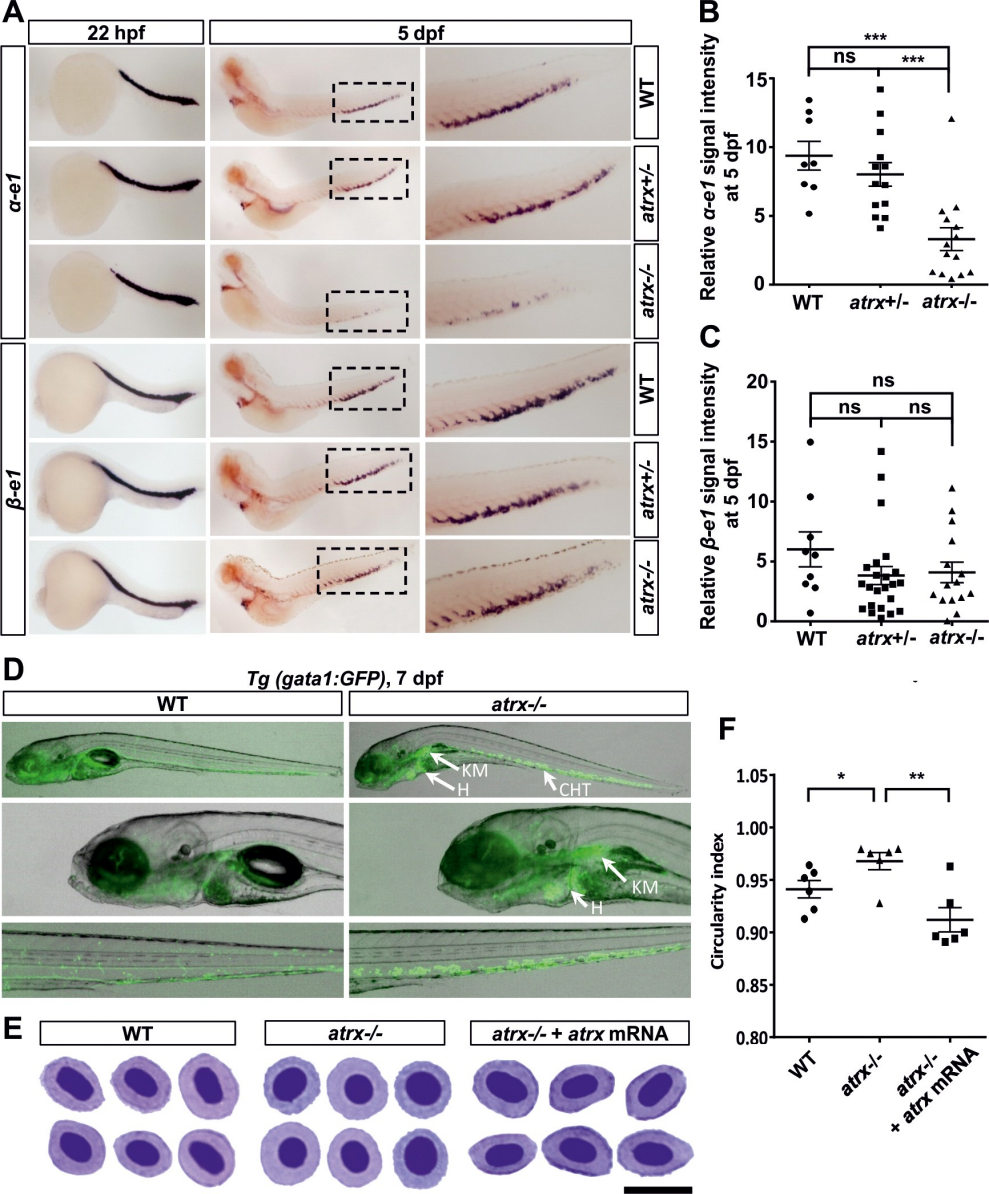
Homozygous loss of *atrx* results in an α-thalassemia-like phenotype in zebrafish development. **(A)** Whole-mount *in situ* hybridization for *α-e1* and *β-e1 globins* in wildtype (WT), *atrx*+/- heterozygous fish and *atrx*-/- homozygous mutants at 22 hpf and 5 dpf as indicated. Boxes outline the CHT region at 5 dpf, and are magnified in the right panels. Globin signal intensities of *α-e1* **(B)** and *β-e1* **(C**) in fish with different *atrx* backgrounds were calculated at 5dpf. Horizontal bars indicate the means ± SEM, which were compared with the two-tailed unpaired *t*-test; ns = not significant; ***p<0.001. **(D)** Erythroid progenitors development visualized by GFP in the *Tg(gata1*:*GFP)* transgenic line with wildtype (WT) or *atrx*-/- background at 7 dpf. CHT = caudal hematopoietic tissue; H = heart; KM = kidney marrow; dpf = days post fertilization. **(E)** Analysis of peripheral blood smears by MGG staining in wildtype fish (WT), *atrx*-/- homozygous mutant and *atrx*-/- homozygous mutant injected with zebrafish *atrx* mRNA at 7 dpf; scale bar: 10 μm. **(F)** Circularity index for the erythroid cells from fish with different *atrx* backgrounds **(E)** was calculated by ImageJ. Horizontal bars indicate the means ± SEM, which were compared with the two-tailed unpaired *t*-test; *p<0.05; **p<0.01.

Previous reports from human patients have shown that *ATRX* mutations are associated with reduced *α-globin* expression and lead to α-thalassemia myelodysplasia syndrome (ATMDS) [1–5]. To investigate the *globin* expression in *atrx* mutant zebrafish, we performed whole-mount *in situ* hybridization (WISH) using *α-e1* and *β-e1 globin* probes. The results showed that the expression levels of *α-e1 globin*, but not *β-e1 globin*, were significantly reduced in *atrx*-/- homozygous mutants compared to *atrx*+/- heterozygous and wildtype siblings. This was observed during definitive erythropoiesis at 5 dpf, but not during primitive erythropoiesis at 22 hours post fertilization (hpf) (Fig 2A–2C), analogous to findings in humans [1]. Normal *globin* gene expression during primitive erythropoiesis likely reflects presence of maternal RNA or protein [36]. The expression levels of *c-myb* at both 36 hpf and 5 dpf did not show significant differences between *atrx*-/- homozygous mutants and wildtype embryos (S2A–S2C Fig), indicating that the development of hematopoietic stem/progenitor cells was not affected in *atrx*-/- homozygous mutants.

To study the effects of reduced globin expression on erythropoiesis, we bred the zebrafish *atrx*+/- mutant with the *Tg(gata1*:*GFP)* transgenic line [37]. In this line, GFP expression is driven by the zebrafish *gata1* promoter, providing a useful marker to identify erythroid cells in the *Tg(gata1*:*GFP)* transgenic line. At both 7 dpf and 12 dpf, *atrx*-/- homozygous mutants showed a remarkable increase in GFP-expressing cells in the heart, kidney marrow and caudal hematopoietic tissue (CHT) compared to wildtype fish (Figs 2D and S2D), indicating the accumulation of erythroid progenitors in these regions. Furthermore, May-Grunwald-Giemsa (MGG) staining of peripheral blood smears from *atrx*-/- homozygous mutants showed that erythrocytes had an aberrant rounded shape compared with the characteristic flattened elliptical morphology observed in the wildtype fish at 7 dpf. Zebrafish *atrx* mRNA injection rescued the mutant phenotype (Fig 2E and 2F). The rounded erythrocytes indicate the presence of circulating erythroid progenitors in the *atrx*-/- homozygous mutants, reflecting a block in erythroid cell differentiation resulting from the lack of *α-globin* expression. Taken together, these data indicate that the zebrafish *atrx* knockout model closely resembles the phenotype of human thalassemia patients with *ATRX* mutation, showing reduced *globin* expression and the accumulation of erythroid progenitors [1].

### Additional loss of *atrx* in *p53/nf1-*deficient zebrafish promotes initiation of specific tumor types

Because *ATRX* is a tumor suppressor in many types of human cancers [13–19], we examined the oncogenic effects of haploinsufficiency for *artx* alone and in cooperation with the *p53/nf1*-deficient genetic background. Both, *p53* and *NF1* are known tumor suppressors in humans and are inactivated in various cancer types. Loss of NF1 removes a major source of GTPase-activation affecting RAS and thus prolongs and strengthens RAS-MAPK signaling, thus enhancing the proliferation and survival of tumor cells [38,39]. The loss of *nf1* has previously been shown to synergize with *p53* mutation in a zebrafish model of MPNSTs and high-grade gliomas [29]. In zebrafish, the *nf1* gene is duplicated (*nf1a* and *nf1b*) resulting in four functional *nf1* alleles. Since a complete loss of *nf1* is lethal, we bred *p53-/-;nf1b-/-;nf1a+/-* fish, resulting in offspring with the *p53-/-;nf1b-/-;nf1a+/-* or *p53-/-;nf1b-/-;nf1a+/+* genotypes. Zebrafish with both genotypes are prone to develop MPNSTs, but with much faster onset and increased penetrance in the *nf1a+/-* genotype fish [29]. Moreover, we have previously observed high grade glioma tumorigenesis in *p53-/-;nf1b-/-;nf1a+/-* fish arising with low penetrance. Using CRISPR/Cas9, we created the *atrx*+/- genotype in the *p53-/-;nf1b-/-;nf1a+/-* background. Since a total loss of both *nf1* and *atrx* is lethal in development, in-cross of this line resulted in viable offspring with 4 genotypes, 1) *p53-/-;nf1b-/-;nf1a+/-;atrx+/-*, 2) *p53-/-;nf1b-/-;nf1a+/+;atrx+/-*, 3) *p53-/-;nf1b-/-;nf1a+/-;atrx+/+*, and 4) *p53-/-;nf1b-/-;nf1a+/+;atrx+/+*.

**Fig 3:**
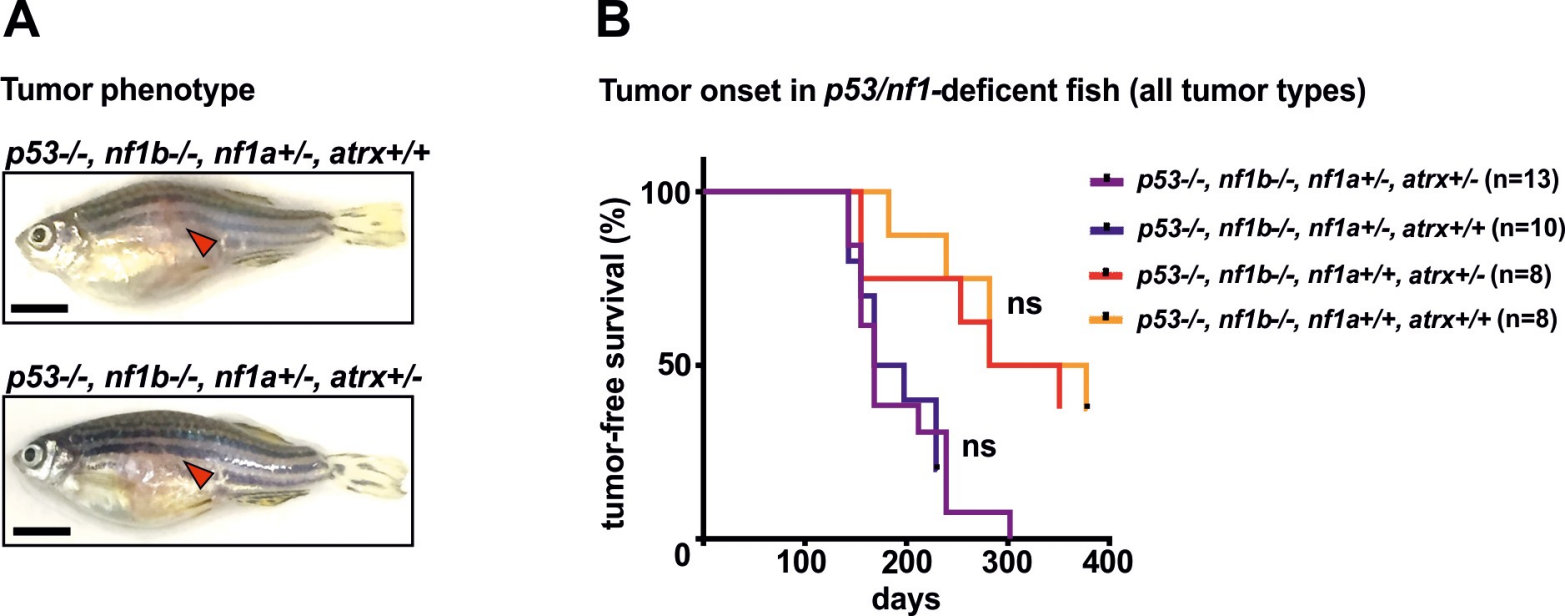
Addition of frameshift-mutations in *atrx* to the *p53/nf1*-deficient background induces specific changes in tumor biology. **(A)** The morphologic features of *p53/nf1/atrx-* and *p53/nf1*-deficient tumors were indistinguishable by visual inspection. **(B)** When all tumor types were considered, tumor onset was not significantly altered upon heterozygous loss of *atrx*; a representative tumor watch experiment using line 1 is shown; the tumor watch has been reproduced in lines 1 and 2 with similar results; ns = not significant.

Fish of all these genotypes were carefully monitored for tumor onset. Both, *atrx*+/+ and *artx*+/- fish of this line developed visually identical tumors located in the eye, gill, head, tail and predominantly in the abdomen (Fig 3A). Surprisingly, tumor watch experiments revealed no differences in time of tumor onset and penetrance associated with the *atrx*+/- genotype (Fig 3B). However, histopathology analysis confirmed that 100% of the tumor bearing *p53-/-;nf1b-/-;nf1a+/-;atrx*+/+ and *p53-/-;nf1b-/-;nf1a+/+;atrx*+/+ control fish had MPNSTs (n = 21 and n = 14 respectively; Tables 1 and S2). In the *atrx*+/- siblings of both *nf1a*+/- and *nf1a*+/+ populations, MPNSTs were identified in between 83.3% and 97.1% of all tumor-bearing fish and were indistinguishable in histology from their *atrx+/+* control counterparts (n = 34 and n = 12, respectively; S3A Fig and Tables 1 and S2). Because ATRX is known to influence PRC2-mediated gene silencing, we examined lysine 27 tri-methylation status of histone 3 (H3K27me3), which is an epigenetic modification associated with genes silenced by PRC2, and predicts a worse prognosis for patients when lost in MPNST tissue [40]. Immunofluorescence staining using an H3K27me3-specific antibody revealed that this epigenetic mark was clearly present in *atrx*+/- and *atrx*+/+ tumors in the *p53/nf1*-deficient background (S3B Fig). Thus, partial *atrx* loss did not appear to have an inhibitory effect on total H3K27me3 deposition.

**Table 1:** Tumor spectrum of *p53/nf1/atrx*-deficient and control fish. Individual fish have tumors of multiple organs with different histology, so that the number of tumors can exceed the number of fish.

***p53-/-*, *nf1b-/-*, *nf1a+/+*, *atrx+/-***	n = 12		
**Tumor type**	**Onset at age (days)**	**Total number**	**% in fish**
MPNST	189–377	10 out of 12	83.3
Epithelioid sarcoma	189	1 out of 12	8.3
Undifferentiated pleomorphic sarcoma	377	1 out of 12	8.3
Papillary serous carcinoma	421	1 out of 12	8.3
***p53-/-*, *nf1b-/-*, *nf1a+/+* controls**	n = 14		
**Tumor type**	**Onset at age (days)**	**Total number**	**% in fish**
MPNST	216–377	14 out of 14	100.0
T-ALL	265–364	2 out of 14	14.3
***p53-/-*, *nf1b-/-*, *nf1a+/-*, *atrx+/-***	n = 34		
**Tumor type**	**Onset at age (days)**	**Total number**	**% in fish**
MPNST	138–393	33 out of 34	97.1
Epithelioid sarcoma	220–288	2 out of 34	5.9
Differentiated angiosarcoma	174	1 out of 34	2.9
Undifferentiated blue cell tumor	140	1 out of 34	2.9
Biliary cancer	138	1 out of 34	2.9
T-ALL	393	1 out of 34	2.9
***p53-/-*, *nf1b-/-*, *nf1a+/-* controls**	n = 21		
**Tumor type**	**Onset at age (days)**	**Total number**	**% in fish**
MPNST	133–307	21 out of 21	100.0

**Fig 4:**
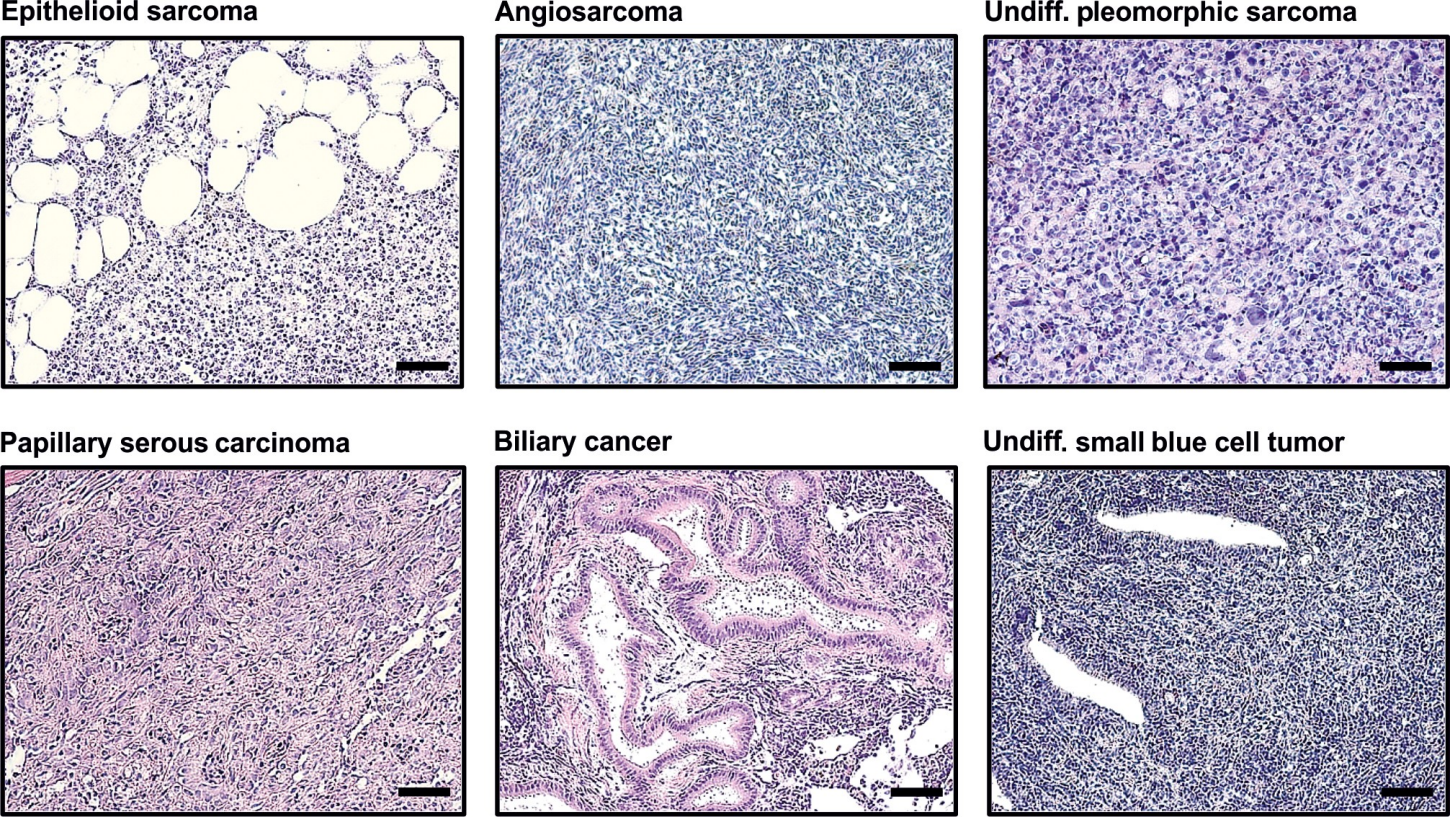
Histology of tumor types detected in *atrx*+/- zebrafish with *p53/nf1*-deficient background. This figure shows HE-stained sections of all tumor types detected in *p53-/-*, *nf1b-/-*, *nf1a+/-*, *atrx+/-* or *p53-/-*, *nf1b-/-*, *nf1a+/+*, *atrx+/-* fish that were not observed in *atrx*-wildtype siblings; scale bars: 50μm.

**Fig 5:**
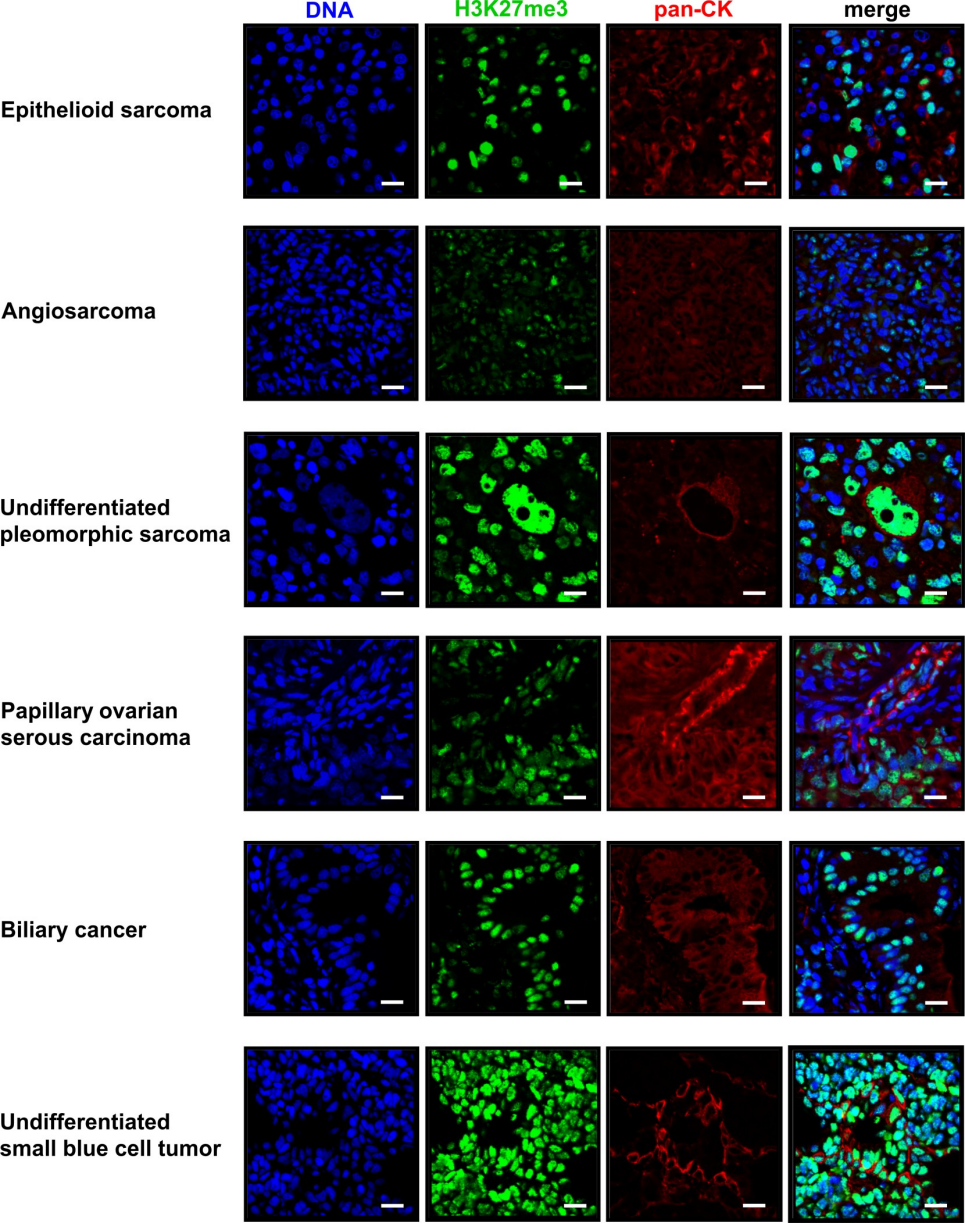
Immunofluorescence staining of tumor types detected in *atrx*+/- zebrafish with *p53/nf1*-deficient background. This figure shows all tumor types detected in *p53-/-*, *nf1b-/-*, *nf1a+/-*, *atrx+/-* or *p53-/-*, *nf1b-/-*, *nf1a+/+*, *atrx+/-* fish that were not observed in *atrx*-wildtype siblings. For each tumor a section stained by indirect immunofluorescence for H3K27me3 (green) and pan-cytokeratin (red) is shown. The DNA is visualized with Hoechst (blue); scale bars: 10μm.

Histopathologic analysis further revealed that 5 out of 34 (14.7%) analyzed tumor-bearing fish of the *p53*-/-;*nf1b*-/-;*nf1a*+/-;*atrx*+/- population had various tumor types other than or in addition to MPNSTs, including epithelioid sarcoma, biliary cancer, angiosarcoma, and an undifferentiated tumor of the eye with small round blue cell morphology (Table 1 and Fig 4). In the *p53-/-;nf1b-/-;nf1a+/+;atrx+/-* population this proportion was higher, with 3 out of 12 (25%) of zebrafish developing neoplasms consistent in histology with epithelioid sarcoma, undifferentiated pleomorphic sarcoma, or serous carcinoma of the ovary. These tumor types were never observed in *atrx*-wildtype siblings. It is noteworthy that many individual fish harbored more than one tumor site or type at the time of sacrifice. Epithelioid sarcomas were identified by the histologic appearance and positive cytokeratin (CK) staining of sections from paraffin embedded tumor tissue using the pan-CK antibody AE1/AE3 which is known to positively stain the vast majority of epithelioid sarcomas [41]. CK expression was also found in papillary serous carcinoma, undifferentiated pleomorphic sarcoma and biliary cancer (Fig 5). Faint positive staining for CK was detected in angiosarcoma tissue. All non-MPNST tumors in *atrx+/-* fish also stained strongly positive for the H3K27me3 mark (Fig 5). All detected sarcoma types other than MPNSTs are summarized below as soft tissue sarcomas.

In previous studies, *ATRX* was identified as a tumor suppressor gene involved in the pathogenesis of gliomas, sarcomas and neuroendocrine tumors [13–19]. Moreover, in the AACR Genie database, *ATRX* mutations are annotated in 49 cancer type categories (S3 Table), representing at least 45 distinct malignancies [32]. Within these, uterine sarcoma and glioma have the highest proportion of *ATRX*-mutant samples (19.60% and 16.74% respectively), whereas soft tissue sarcomas are ranked 15^th^ with 6.42%. Moreover, the *ATRX*-mutation frequency in ovarian cancer is 2.65%, and 1.12% in hepatobiliary carcinoma. Thus, the tumor types observed in our model of combined *p53/nf1/atrx*-deficiency are faithfully reflected in the human *ATRX-*mutant tumor spectrum.

**Fig 6:**
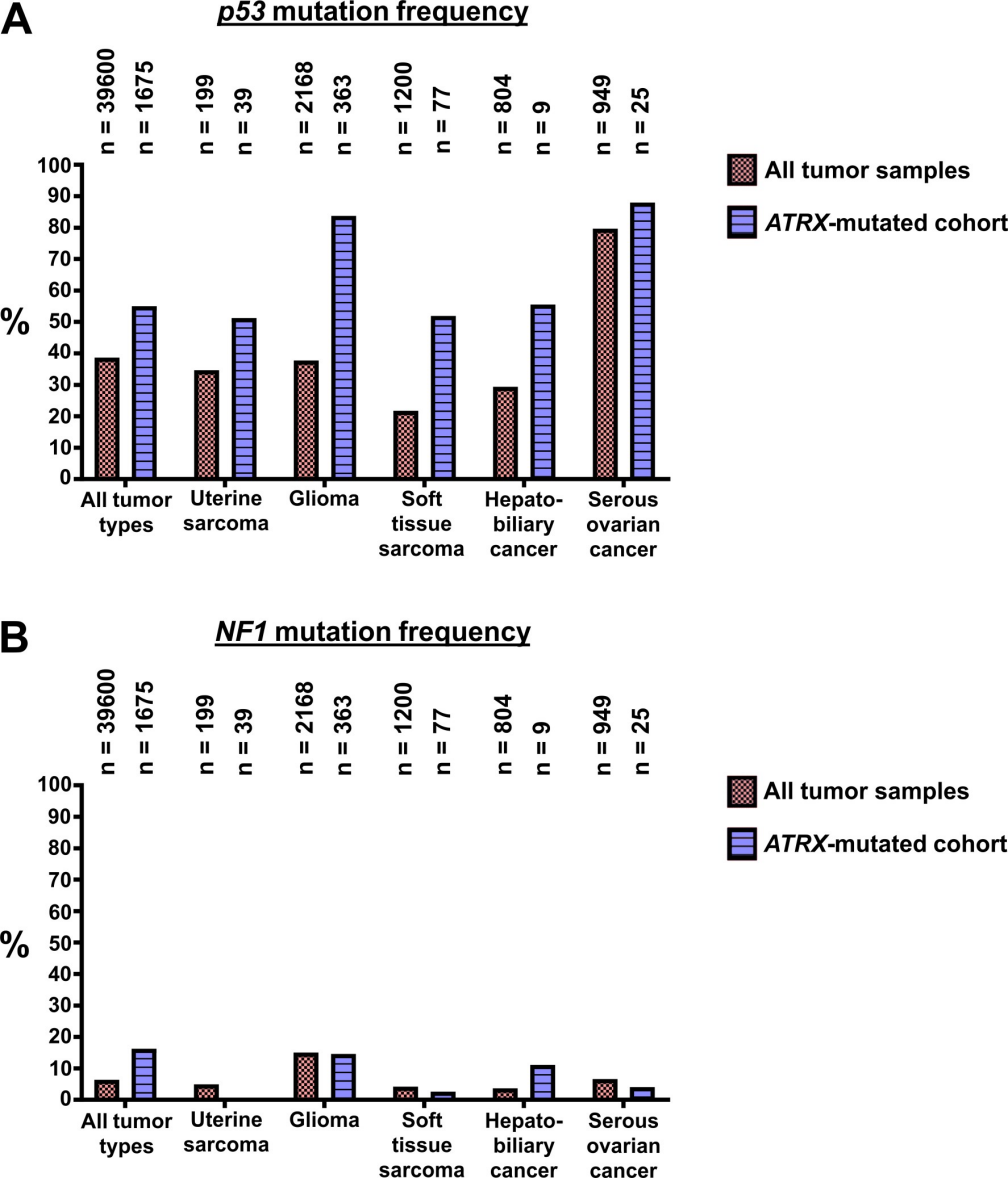
AACR Genie data on the co-occurrence of mutations in *ATRX* and *p53* or *NF1* in selected tumor types. Data on mutation frequencies in *p53* and *NF1* were extracted from the AACR Genie Database [32].

Soft tissue sarcomas and the other tumor types depicted in Fig 4 were not observed in the *atrx-*wildtype, *p53/nf1-*deficient controls or in the *atrx*+/-, *p53/nf1-*wildtype line. Thus, the *atrx*+/- genotype cooperated with the *p53/nf1*-deficient background in the development of these malignancies. Interestingly, *p53/nf1/atrx*-mutant fish developed various other tumor types regardless of whether two or three *nf1* alleles were mutated (Tables 1 and S2), indicating *atrx*-deficiency synergizes with the combined loss of *p53* rather than *nf1*. This cooperation between lof in *p53* and *atrx* in our model is supported by previous data on the combined lof mutations in *ATRX* and *p53* in high-grade gliomas [13] and leiomyosarcomas [19]. In the AACR Genie database, the overall *p53* mutation rate considering all tumor samples analyzed is 38.64%, whereas among the *ATRX*-mutant samples 55.04% are co-mutants for *p53* (Fig 6A) [32]. Gliomas, the tumors with the second highest prevalence of *ATRX*-mutation in the database (S3 Table) carry about twice as often a mutation in *p53* if they have a mutation in *ATRX* (Fig 6A). In *ATRX*-mutant soft tissue sarcomas *p53* mutations are observed almost 2.5 times as often as in the overall cohort. Together, these relationships indicate a selection advantage during the development of these particular tumor types for malignant clones with combined deficiencies in *ATRX* and *p53*. This does not extend as clearly to the loss of *NF1* in soft tissue sarcomas, gliomas, and serous ovarian cancer (Fig 6B). However, when again all tumor samples were considered, similar results as for *p53* were observed with 6.4% *NF1*-mutation rate in all tumor samples and 16.28% in the *ATRX*-mutated cohort. This indicates that both, loss of *p53* and *NF1*, might cooperate with *ATRX*-deficiency in the right cancer type and genetic background. In hepatobiliary cancer in particular, there is a threefold higher incidence of *nf1* mutations in *atrx*-mutant samples (Fig 6B). Thus loss of *nf1b*, and therefore RAS-MAPK pathway activation, appears to synergize with *atrx* deficiency in this cancer type. In tissues with predominate expression of *nf1b* compared to *nf1a*, the difference between loss of one allele or retention of both alleles could be very significant in terms of *nf1* activity.

Despite the shift to more heterogeneous tumor types, we did not see an accelerated overall tumor onset in *p53/nf1/atrx*-deficient fish compared to *p53/nf1*-knockout control fish, and the proportion of MPNST-bearing fish decreased in the *atrx*+/- cohort. From this result, we conclude that *atrx* loss does not promote the pathogenesis of MPNSTs in our model. In the AACR Genie database *ATRX* mutations are annotated for 1 of 31 analyzed MPNSTs (3.23%), while 2 of 29 samples that were profiled for copy number alterations (CNAs) show a deletion of *atrx* (6.9%). However, CNAs can occur due to general genomic instability and do not have to be specific for a gene contained in a larger deletion. Thus, we cannot exclude the possibility that *ATRX* loss can contribute to MPNST malignancy in the right context (e.g. by supporting ALT), although it clearly it does not accelerate MPNST onset in the genetic background of our model. The observation of an undiminished H3K27me3 signal in MPNSTs upon heterozygous *atrx* knockout in our model fits well to the fact that patients with MPNSTs retaining the H3K27me3 mark have a better prognosis [42].

### Partial loss of *atrx* deregulates *tert* expression and PRC2-mediated gene silencing

**Fig 7:**
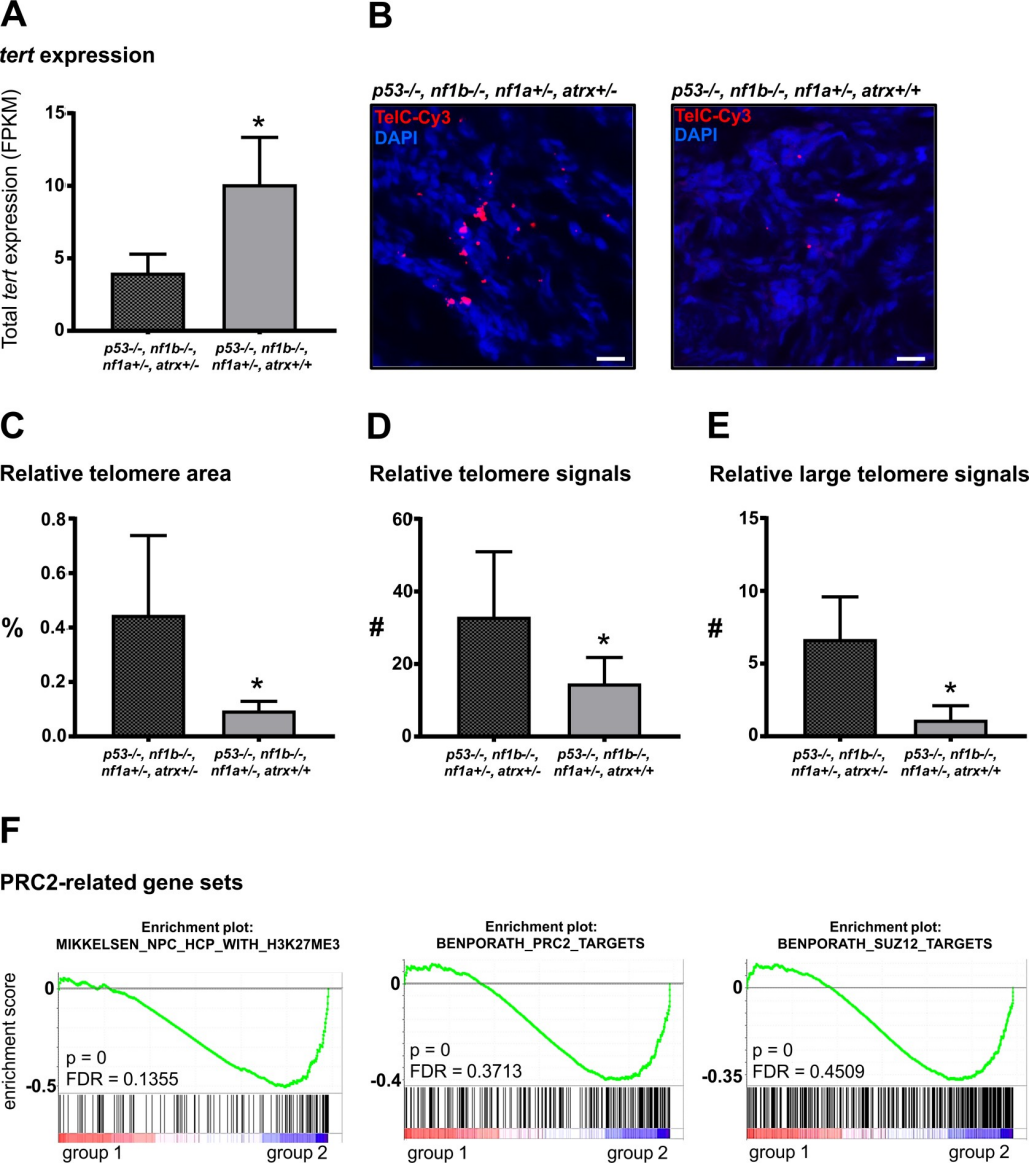
Loss of *atrx* in *p53/nf1*-depleted zebrafish tumors results in *tert* downregulation, ALT and disturbed PRC2 function. **(A)** FPKM values show a significant downregulation of tert in *p53/nf1/atrx*-deficient fish compared to *p53/nf1*-kockout control fish; values were compared with the two-tailed unpaired *t*-test; n = 3, p = 0.0387. **(B)** Fluorescence *in situ* hybridization (FISH) using a Cy3-conjugated telomere-specific probe (TelC-Cy3, red) reveals elongated telomeres visible as large irregularly shaped spots in tumors with a knockout of *p53*, *nf1* and *atrx* compared to *atrx*-wildtype control tumors, consistent with alternative lengthening of the telomeres; DNA stained with DAPI (blue); scale bars: 10μm; n = 6. **(C)** Quantification of relative telomere area in FISH images, normalized to DAPI signal area; values were compared with the two-tailed unpaired *t*-test; *p = 0.0151; n = 6. **(D)** Quantification of the relative number (#) of all detectable telomere spots in FISH images, normalized to DAPI signal area; values were compared with the two-tailed unpaired *t*-test; *p = 0.042; n = 6. **(E)** Quantification of the relative number (#) of telomere spots larger than ~1.5μm^2^ in FISH images, normalized to DAPI signal area; values were compared with the two-tailed unpaired *t*-test; *p = 0.0013; n = 6. **(F)** RNA-seq analysis demonstrates significantly increased expression of PRC2-target gene sets in *atrx*-deficient tumors; group 1: *p53-/-*, *nf1b-/-*, *nf1a+/-*, *atrx+/+* (control); group 2: *p53-/-*, *nf1b-/-*, *nf1a+/-*, *atrx+/-*; n = 3.

The soft tissue sarcomas and other tumor types detected in *p53/nf1/atrx-*deficient fish were never seen in *atrx* wildtype siblings and have not been described in previous genome-editing studies in the *p53/nf1*-deficient line [29,43]. Thus, we conclude that the onset of these tumors was caused by decreased ATRX activity associated with the *atrx*+/- genotype. To examine the effect of heterozygous loss of *atrx* on global gene expression we used mRNA isolated from zebrafish tumor tissue to compare the gene expression profiles of *p53-/-;nf1b-/-;nf1a+/-;atrx+/-* and *p53-/-;nf1b-/-;nf1a+/-;atrx+/+* MPNSTs by RNA-seq with three biological replicates for each group. Strikingly, the telomerase encoding gene *tert* was significantly downregulated in *atrx+/-* tumor tissue (Figs 7A and S4 and S4 Table). Since ATRX loss in humans is associated with ALT, we used fluorescence *in situ* hybridization with a telomere-specific probe (TelC-FISH) to visualize the telomeres of *atrx+/-* and *atrx+/+* tumors in our line. In this experiment, we detected an increased telomere signal consistent with previously described phenotypes of ALT in human tumors (Fig 7B) [13]. This indicates that longer telomeres associated with *atrx* depletion-mediated ALT triggers a downregulation of *tert*. Indeed, a negative feedback mechanism that regulates *TERT* expression in a telomere length-dependent manner has been previously described in human cancer cells [44], and is supported by our observations to occur in our model system. Quantification of the telomere FISH-signal (n = 6) relative to the DNA-signal revealed a significantly larger total telomere area in TelC-FISH images derived from *atrx*+/- tumors compared to images derived from *atrx*+/+ controls (Fig 7C and S5 Table; p = 0.0151). Moreover, the total number of detectable telomere spots also increased significantly (Fig 7D and S6 Table; p = 0.042). This effect was even stronger, when only larger telomere signals (>1.5μm^2^ area) were considered (Fig 7E and S6 Table; p = 0.0013). This shows that heterozygous loss of *atrx* resulted in a measurable increase in telomere size which indicated *atrx*-deficiency-related ALT.

In adult zebrafish tissues, the Tert protein regulates aging in a process similar to its role in human cells [45–47]. In a previous study, *tert-/-* zebrafish aged faster and developed earlier spontaneous age-related tumors [46]. Thus, in zebrafish, shortening of the telomeres promotes tumor onset, which can be explained by the need in early tumorigenesis to increase the mutational burden by genomic instability in order to develop a malignant cancer. In patients, similar findings were reported for the transition of adenomas to metastatic colon cancer, which is among the most well described cancer progression systems. In particular, cells within adenomas that give rise to metastatic cancer have critically short telomeres [48]. In this context, induction of ALT due to the loss of *atrx* would not be expected to accelerate the onset of tumors, but rather facilitate later phases of their progression. This may explain why the tumor onset was not uniformly accelerated in our model. Thus, ALT cannot alone be responsible for the *atrx* lof-mediated onset of various tumor types including soft tissue sarcomas detected in our model.

To identify factors that may have contributed to this carcinogenesis we analyzed mRNA-seq data for gene set enrichment that might contribute to tumor formation. In this survey, we found the enrichment of notch1-targets (one gene set) and jak-stat-signaling (one gene set), epithelial differentiation (three gene sets) and PRC2-function (11 gene sets) in *p53/nf1/atrx*-deficient tumors compared to the *atrx* wildtype controls (Figs 7F and S5, S7 and S8 Tables). Interestingly, 10 of 11 PRC2-related gene sets showed a significant up-regulation of PRC2-targets, SUZ12-targets or H3K27me3 marked genes from various stem and progenitor cell types. However, there was one EZH2-target gene set that was significantly downregulated in *p53/nf1/atrx*-depleted tumors. This indicates that the loss of *atrx* predominantly led to a re-expression of PRC2 target genes silenced by H3K27me3 deposition, even though this might not affect all PRC2-targets. Interestingly, this was accompanied by an undiminished nuclear H3K27me3 signal in *p53/nf1/atrx*-deficient tumors detected by immunofluorescence. This observation is consistent with a previous study in human cells where loss of *ATRX* was found to displace PCR2-deposited H3K27me3 silencing marks away from the target gene promoters to the intergenic space leading to their re-expression [9]. Thus, it is likely that ATRX loss does not abolish H3K27me3 marks in the genome, but rather deregulates their positioning throughout the genome.

In this study, we show the consequences of inactivation of *atrx* in the zebrafish germline, which resulted in the first zebrafish model *atrx* deficiency causing alpha-thalassemia and the contribution of loss of *atrx* to carcinogenesis in the background of *p53* loss and RAS-MAPK pathway activation. Faithful models of both of these consequences of *atrx* loss will be valuable for future preclinical studies. We provide evidence that *atrx* and *p53* cooperate in the carcinogenesis of soft tissue sarcomas and other malignancies. Our data are consistent with a negative feedback loop downregulating telomerase upon loss of *atrx*, causing alternative lengthening of the telomeres, and indicate that the role of *atrx*-deficiency in tumor initiation may also be linked to disturbed PRC2-mediated gene silencing.

## Materials and methods

### Ethics statement

All zebrafish studies and maintenance were done in accord with Dana-Farber Cancer Institute IACUC-approved protocol (#02–107).

### Zebrafish lines and maintenance

Zebrafish were raised and maintained according to standard procedures. They were derived from the AB background strain, the *Tg(gata1*:*GFP)* line [37] or the *p53/nf1*-deficient background [29,49]. All *p53*-/- fish were homozygous for the *p53*-M214K mutation described previously [49]. The *p53/nf1*-deficient fish carried a frameshift mutation in exon 26 of *nf1a* and in exon 17 of *nf1b* which truncate the Nf1 protein before its functional GRD domain, as published in a previous study [29].

The zebrafish *atrx* mutant lines were generated by the CRISPR/Cas genome editing system using the previous described protocol [30]. The plasmid constructs pDR274 (#42250) and pMLM3613 (#42251) were purchased from Addgene. Oligonucleotides 5’-TAG GTC CTG AGT TCC GTA ACA A-3’ and 5’-AAA CTT GTT ACG GAA CTC AGG A-3’ were annealed and cloned into the pDR274 vector to generate single guide RNAs (gRNAs) targeting *atrx* exon 4. Each embryo was injected with 1 nl of solution containing 25 ng/μl gRNA and 600 ng/μl *Cas9* mRNA at the 1 cell stage. Mosaic F0 fish with germline mutation were identified, and stable mutant lines were established by outcrossing. To genotype the *atrx* mutant line, we first amplified a DNA fragment containing the mutated site, using the primer pair *atrx*_E3I3Fw: 5’-GCA AGC TGC CAC AAG GTT AGT CC-3’ and *atrx*_I4Rv: 5’-GTC ACA AAC ACG TCA CCA CTT A-3’ with genomic DNA extracted from fin clips serving as template. The DNA product was either sent for sequencing with the primer *atrx*_seqI3Fw: 5’-TGT TCC GAT CAG TCT TCC TTA GC-3’ or digested by BslI. The wildtype DNA fragment was then digested into 2 fragments of 171 (bp) and 282 bp while the mutant one was resistant to digestion, revealing a band of 455 to 475 bp (please note that the mutant PCR product can be larger due to insertions). Alternatively, if PCR products were not sequenced, primers *atrx*_shortFw: 5’-GCAGGCACAGTAGTGGTGAAGCCA-3’ and *atrx*_shortRv: 5’- CACCAGGACGTTTCCGCGCACCCT-3’ were used. The wildtype DNA fragment was then digested into 79 and 45bp fragments. The mutant amplicon remained undigested at a size of 126 to 146 bp (please note that the mutant PCR product can be larger due to insertions).

### Injection in 1-cell-stage embryos

All gRNA was transcribed *in vitro* from DraI (NEB) linearized pDR274-gRNA plasmid DNA using the MAXIscript T7 Kit (Ambion Inc.). *Cas9* mRNA was transcribed *in vitro* from NotI (NEB, Ipswich, MA, United States) linearized pCS2-nCas9n plasmid DNA using the mMessage mMachine SP6 Kit (Ambion Inc., Foster City, CA, United States). Oligonucleotides were mixed between 1:5 and 1:1 with a 0.5% phenol red solution (Sigma-Aldrich, Burlington, MA, United States) to a final concentration of 25ng/μl gRNA and 600ng/μl *Cas9* mRNA. To induce mutations in the genome of zebrafish, we injected one-cell-stage embryos with the oligonucleotide/phenol red mix described above within 30min after fertilization using a glass capillary mounted into an air pressure injector (Harvard Apparatus, Cambridge, MA, United States). The injection volume was 1nl oligonucleotide/phenol red mix per embryo. Dead embryos were removed 3 to 6 hours after injection using a Leica M420 microscope (Wetzlar, Germany). For zebrafish *atrx* rescue experiment, *atrx* was cloned into pCS2+ vector by PCR from cDNA of zebrafish embryos, using the primer pair *atrx*_Fw: 5’-CGG CTC GAG ATG GCA ACC AAT GAC GTA AAT ATT-3’ and *atrx*_Rv: 5’-CGG TAC GTA TTA CAG ACC CTT AGA TGG GCC TGG-3’. Zebrafish *atrx* mRNA was synthesized *in vitro* from NotI (NEB, Ipswich, MA, United States) linearized *atrx*-pCS2+ plasmid DNA using the mMessage mMachine SP6 Kit (Ambion Inc., Foster City, CA, United States). 1 nl *atrx* mRNA (500 ng/μl) containing phenol red dye was injected into zebrafish embryos at the one-cell stage.

### Tumor watch in tumor-prone zebrafish lines

The *sox10*:GFP zebrafish were genotyped at 2–3 months of age and separated into distinct tanks according to genotype. At least every 2 weeks, all fish were examined for tumor onset using a Nikon C-DSD115 fluorescence microscope (Tokyo, Japan). The time point of tumor onset was defined at the first observation of a GFP+ growth that did not regress within 2 weeks. Fish that died from tumor-unrelated causes were removed from the analysis. A survival analysis with Graph pad Prism 7 software was performed to visualize the tumor onset rate and tumor penetrance.

### Zebrafish histopathology

Tumor-bearing fish were sacrificed and fixed in 4% paraformaldehyde (PFA) in PBS at 4°C for 1–3 days. Subsequently, the fish were washed in PBS or 70% ethanol and embedded in paraffin. Paraffin sectioning (3-micron) and hematoxylin/eosin staining were performed at the DF/HCC Research Pathology Core according to standard protocols.

### Indirect immunofluorescence of paraffin-embedded tumor tissue

The indirect immunofluorescence protocol was adapted from previous studies [50,51]. Zebrafish tumor sections were incubated twice for 10 min each in xylol to remove paraffin. For rehydration the slides were washed for 5min each in 100% ethanol, 96% ethanol, 70% ethanol twice for 5min each in H_2_Odd. Epitopes were unmasked by heating for 2min and 30 sec in 10 mmol citric acid/NaCitrate-buffer (pH 6; 18% citric acid, 82% NaCitrate) followed by 5min incubation after each cooking step. Next, slides were blocked for 15min in PBS + 0.1% BSA at room temperature. The DNA was stained for 10 min with Hoechst 33342 (1mg/ml) and diluted 1:250 diluted in PBS + 0.1% BSA. Primary antibodies were diluted in PBS + 0.1% BSA and 100μl antibody-mix was incubated for at least 1h on fat surrounded area around the tissue (wet chamber in the dark). Secondary antibodies were diluted in PBS + 0.1% BSA and 100μl antibody-mix was incubated for 1h on fat surrounded area around the tissue (wet chamber in the dark). Slides were washed for 5-10min in PBS + 0.1% BSA after each staining step. After secondary antibody incubation the slides were washed 1x in PBS + 0.1% BSA, 1 x in PBS and 1x in H_2_Odd, dried with an absorbent paper and mounted in 30μl PromoFluor Antifade Reagent mounting medium (Promokine, Heidelberg, Germany) using a #1 cover glass. As primary antibodies were used: pan-cytokeratin AE1/AE3 (Novus Biologicals, Littleton, CO, USA), H3K27me3 C36B11 (Cell Signaling Technology, Danvers, MA, USA). Secondary antibodies were conjugated with Alexa 488, 568 (Thermo Fisher Scientific, Waltham, MA, USA). Slides were imaged by a Leica SP5X scanning confocal microscope (Wetzlar, Germany) at the Confocal and Light Microscopy core facility at Dana-Farber Cancer Institute.

### Telomere PNA-fluorescence *in situ* hybridization (FISH)

Telomere PNA-FISH was performed as previously described [47]. Briefly, zebrafish paraffin sections were deparaffinized, followed by hybridization for 2 h at room temperature in the dark. Slides were washed, mounted and imaged by a Leica SP5X scanning confocal microscope at the Confocal and Light Microscopy core facility at Dana-Farber Cancer Institute. Cy3 labeled PNA TelC probes (CCCTAA repeats) were purchased from PNA Bio INC (Thousand Oaks, CA, USA). DNA was counterstained with DAPI. Individual images of the Cy3 and DAPI channels were analyzed in ImageJ to quantify the total area covered by Cy3 stained telomeres or DNA and the number of telomere signals (particles in Cy3 channel). Moreover, the average number of large telomere spots was quantified to determine changes in the abundance of large telomeres reminiscent of alternative lengthening of the telomeres. Large was defined as >0,01 inch^2^ size in digital image exported from LasX software (Leica) as single channel image which corresponds to ~1,556μm^2^ real size. For each image these values were normalized to the DNA content of the respective image to quantify the relative telomere signal within the nuclear compartment. For this the area percentage value of the DAPI signal in each image was determined using ImageJ. From this a “DNA content factor” was calculated by the formula ^100^/_% DNA content_. Raw values were subsequently multiplied with this DNA content factor. For each analysis the threshold was set to include the specific signal and exclude the background. The threshold was kept constant for all images compared. Images with background signals that could not be eliminated were excluded from the analysis. A two-tailed unpaired *t*-test type 2 was performed using GraphPad Prism software.

### Whole-mount RNA *in situ* hybridization (WISH)

Riboprobe labeling and WISH were performed as described previously [52,53]. The digoxigenin (DIG)-labeled RNA probes to detect *α-e1* and *β-e1* globins [54], and *c-myb* [55] were as previously described.

### Peripheral blood smears and May-Grunwald-Giemsa (MGG) staining

Peripheral blood smears were prepared on glass slides as described previously [55]. The slides were fixed and stained with May-Grunwald–Giemsa (MGG) solution (Sigma-Aldrich, St. Louis, MO) according to the manufacturer’s instructions and visualized with a Zeiss AXIO microscope (Zeiss, Oberkochen, Germany).

### RNA-Seq data analysis

RNA was isolated from one half of *p53/nf1/atrx*-deficient MPNSTs and *p53/nf1-*deficient control MPNSTs using the AllPrep DNA/RNA Mini Kit from Qiagen (Hilden, Germany) and the other half was analyzed by histopathology as described above. Library preparation, quality control, and next-generation sequencing were conducted by the Molecular Biology Core Facility of the Dana-Farber Cancer Institute following standard protocols.

Gene expression values were derived from paired end RNA-Seq data that compared samples from *p53/nf1/atrx*-deficient tumors to samples from *atrx-*wildtype controls (3 vs. 3 samples). FastQC was used to evaluate read quality on raw RNA-Seq reads and trimmed reads. Trimming of low quality reads and clipping of sequencing adapters was done using the program Trimmomatic [56] and all reads shorter than 36bp after trimming were dropped. Reads were aligned to the GRCz10 version of the zebrafish reference genome with TopHat [57] version 2.1.0. Bam sorting and indexing was done with SamTools [58] and duplicate reads were removed using Picard-tools (http://picard.sourceforge.net). Gene level counts were obtained with htseq-count version 0.9.1 [59]. Differential gene expression was evaluated with the R Bioconductor package DESeq2 [60] and normalized expression values for individual samples were obtained from DESeq2 using the variance stabilizing transform on the raw counts. The variance stabilizing transformed data were used for GSEA.

### Gene set enrichment analysis

Gene Set Enrichment Analysis (GSEA) [61,62] was used to evaluate the association of genes with compared *p53/nf1/atrx*-deficient tumors to the *atrx-*wildtype controls. GSEA was run with 2500 permutations of the phenotype using signal-to-noise to rank genes. GSEA was performed with signatures from version 6.0 of the molecular signature database (MolSigDB) (http://www.broadinstitute.org/gsea/msigdb/index.jsp): the c2 curated gene sets from various sources such as online pathway databases, the biomedical literature, and knowledge of domain experts, the c3 motif gene sets, c5 gene ontology (GO) MF, CC, and BP ontologies, and the c7 immunologic signatures.

### AACR Genie database search

Data of the AACR Genie database (version 3.0 public release) were extracted online using the cBioPortal application (http://www.cbioportal.org/genie) and further processed in Microsoft Excel.

## Supporting information

S1 Fig CRISPR/Cas9-induced mutation of the *atrx* coding sequence in zebrafish germline. An exon 4 targeting gRNA was used to induce frameshift mutations in *atrx* coding sequence. Mutations were induced in either wildtype (WT) or *p53/nf1*-deficient genetic background.
(PDF)

S2 Fig Homozygous loss of *atrx* does not affect hematopoietic stem/progenitor cell development. **(A)** Whole-mount *in situ* hybridization for *c-myb* at 36 hpf and 5 dpf in wildtype (WT), *atrx*+/- heterozygous fish and *atrx*-/- homozygous mutants as indicated. Boxes outline the AGM region at 36 hpf and the CHT region at 5 dpf, and are magnified in the right panels. *c-myb* signal intensities at 36 hpf **(B)** and 5 dpf **(C)** in fish with different *atrx* backgrounds were calculated. Horizontal bars indicate the means ± SEM, which were compared with the two-tailed unpaired *t*-test; ns = not significant. **(D)** Erythroid progenitors development visualized by GFP in the *Tg(gata1*:*GFP)* transgenic line with wildtype (WT) or *atrx*-/- background at 12 dpf. AGM = aorta-gonad-mesonephros; CHT = caudal hematopoietic tissue; H = heart; KM = kidney marrow; hpf = hours post fertilization; dpf = days post fertilization.
(PDF)

S3 Fig MPNST biology in *atrx*+/+ and *atrx*+/- siblings in *p53/nf1*-deficient background. **(A)** HE-staining of *p53/nf1 atrx*+/+ and *atrx*+/- MPNSTs reveals no differences in histology; scale bars: 50μm. **(B)** Indirect immunofluorescence staining of two MPNSTs each of the *p53/nf1 atrx*+/+ and *atrx*+/- cohorts both show detectable tri-methylation of histone 3, lysine 27 (H3K27me3, green); scale bars: 10μm.
(PDF)

S4 Fig Head map of RNA-Seq results. Significantly up- or downregulated genes (p<0.025) between *p53-/-*, *nf1b-/-*, *nf1a+/-*, *atrx*+/+ and *p53-/-*, *nf1b-/-*, *nf1a+/-*, *atrx*+/- samples (n = 3). The heat map is row normalized with blue representing minimum expression and red representing maximum expression.
(PDF)

S5 Fig Gene set enrichment analysis. RNA-Seq analysis revealed significantly increased expression of PRC2-related gene sets, NOTCH1 and JAK/STAT signaling targets and markers of epithelial differentiation; group 1: *p53-/-*, *nf1b-/-*, *nf1a+/-*, *atrx+/+* (control); group 2: *p53-/-*, *nf1b-/-*, *nf1a+/-*, *atrx+/-*.
(PDF)

S1 Table Germline *atrx* mutations in F1 in *p53/nf1*-deficient background. (PDF)

S2 Table Histopathology of tumor-bearing fish by age and genotype. (XLSX)

S3 Table Analysis of ATRX-mutation frequency in human malignancies in AACR Genie Database. (PDF)

S4 Table FPKM and log2 FPKM values from cufflinks for atrx +/+ versus atrx +/- samples in p53/nf1-deficient background. (XLSX)

S5 Table Relative quantification of telomere signal spots in FISH images derived from *atrx*+/+ and *atrx*+/- tumors in *p53/nf1*-deficient background. (XLSX)

S6 Table Relative quantification of large telomere signal spots in FISH images derived from *atrx*+/+ and *atrx*+/- tumors in *p53/nf1*-deficient background. (XLSX)

S7 Table Gene sets enriched in *p53-/-, nf1b-/-, nf1a+/-, atrx+/-* MPNSTs. (XLSX)

S8 Table Gene sets enriched in *p53-/-*, *nf1b-/-*, *nf1a+/-* control MPNSTs. (XLSX)
