# Supplementary material for: Loss of atrx cooperates with p53-deficiency to promote the development of sarcomas and other malignancies
Source: PLoS Genet. 2019 Apr 10;15(4):e1008039. doi: 10.1371/journal.pgen.1008039 (PMC6476535; doi:10.1371/journal.pgen.1008039)
Supplement: S1 Fig — An exon 4 targeting gRNA was used to induce frameshift mutations in atrx coding sequence. Mutations were induced in either wildtype (WT) or p53/nf1-deficient genetic background. (PDF) [file pgen.1008039.s001.pdf]

Zebrafish *atrx*

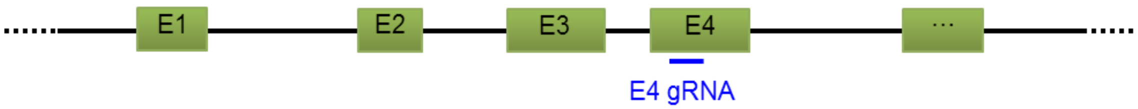

Wildtype background:

atrx exon 4 WT	---GAAACTAAAGATGAATTCCGAGGTCCTGAGTTCCGTAA----CAAGGGAGGCAAAGCGAAAGAAGAGGG---
4in (6in, 2del) (E4)	---GAAACTAAAGATGAATTCCGAGGTCCTGAGTTCCGTGAGTTCCAAGGGAGGCAAAGCGAAAGAAGAGGG---

p53-/-, nf1b-/-, nf1a+/- background:

atrx exon 4 WT	---GAAACTAAAGATGAATTCCGAGGTCCTGAGTTCCGTAA----CAAGGGAGGCAAAGCGAAAGAAGAGGG---	Wildtype
2in (4del, 6in) (E4)	---GAAACTAAAGATGAATTCCGAGGTCCTGAGTTCCTGAGTTCAAGGGAGGCAAAGCGAAAGAAGAGGG---	Line 1
22in (23del/45in) (E4)	---GAAACTAAAGATGAAACTGGTGGTGAAGCCAGAACCAGTTGCTAATGAAACTAAAGATGCCAAGGGAG---	Line 2

S1 Fig: CRISPR/Cas9-induced mutation of the *atrx* coding sequence in zebrafish germline. An exon 4 targeting gRNA was used to induce frameshift mutations in *atrx* coding sequence. Mutations were induced in either wildtype (WT) or *p53/nf1*-deficient genetic background.
