# Supplementary material for: Loss of atrx cooperates with p53-deficiency to promote the development of sarcomas and other malignancies
Source: PLoS Genet. 2019 Apr 10;15(4):e1008039. doi: 10.1371/journal.pgen.1008039 (PMC6476535; doi:10.1371/journal.pgen.1008039)
Supplement: S2 Fig — (A) Whole-mount in situ hybridization for c-myb at 36 hpf and 5 dpf in wildtype (WT), atrx+/- heterozygous fish and atrx-/- homozygous mutants as indicated. Boxes outline the AGM region at 36 hpf and the CHT region at 5 dpf, and are magnified in the right panels. c-myb signal intensities at 36 hpf (B) and 5 dpf (C) in fish with different atrx backgrounds were calculated. Horizontal bars indicate the means ± SEM, which were compared with the two-tailed unpaired t-test; ns = not significant. (D) Erythroid progenitors development visualized by GFP in the Tg(gata1:GFP) transgenic line with wildtype (WT) or atrx-/- background at 12 dpf. AGM = aorta-gonad-mesonephros; CHT = caudal hematopoietic tissue; H = heart; KM = kidney marrow; hpf = hours post fertilization; dpf = days post fertilization. (PDF) [file pgen.1008039.s002.pdf]

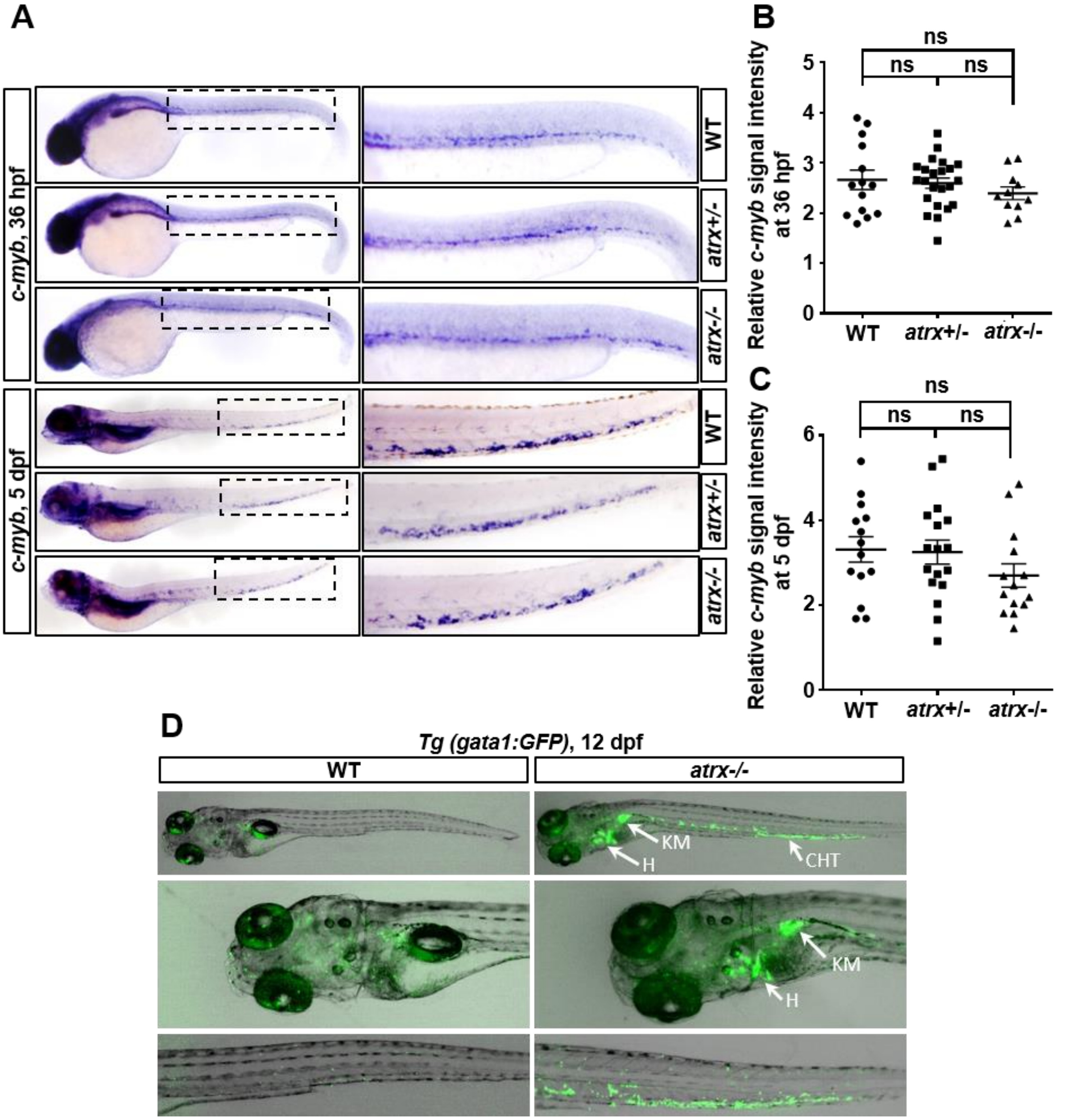

S2 Fig: Homozygous loss of *atrx* does not affect hematopoietic stem/progenitor cell development. (A) Whole-mount *in situ* hybridization for *c-myb* at 36 hpf and 5 dpf in wildtype (WT), *atrx*+/- heterozygous fish and *atrx*-/- homozygous mutants as indicated. Boxes outline the AGM region at 36 hpf and the CHT region at 5 dpf, and are magnified in the right panels. *c-myb* signal intensities at 36 hpf **(B)** and 5 dpf **(C)** in fish with different *atrx* backgrounds were calculated. Horizontal bars indicate the means ± SEM, which were compared with the two-tailed unpaired *t*-test; ns = not significant. **(D)** Erythroid progenitors development visualized by GFP in the *Tg(gata1:GFP)* transgenic line with wildtype (WT) or *atrx*-/- background at 12 dpf. AGM = aorta-gonad-mesonephros; CHT = caudal hematopoietic tissue; H = heart; KM = kidney marrow; hpf = hours post fertilization; dpf = days post fertilization.
