# Supplementary material for: Loss of atrx cooperates with p53-deficiency to promote the development of sarcomas and other malignancies
Source: PLoS Genet. 2019 Apr 10;15(4):e1008039. doi: 10.1371/journal.pgen.1008039 (PMC6476535; doi:10.1371/journal.pgen.1008039)
Supplement: S3 Fig — (A) HE-staining of p53/nf1 atrx+/+ and atrx+/- MPNSTs reveals no differences in histology; scale bars: 50μm. (B) Indirect immunofluorescence staining of two MPNSTs each of the p53/nf1 atrx+/+ and atrx+/- cohorts both show detectable tri-methylation of histone 3, lysine 27 (H3K27me3, green); scale bars: 10μm. (PDF) [file pgen.1008039.s003.pdf]

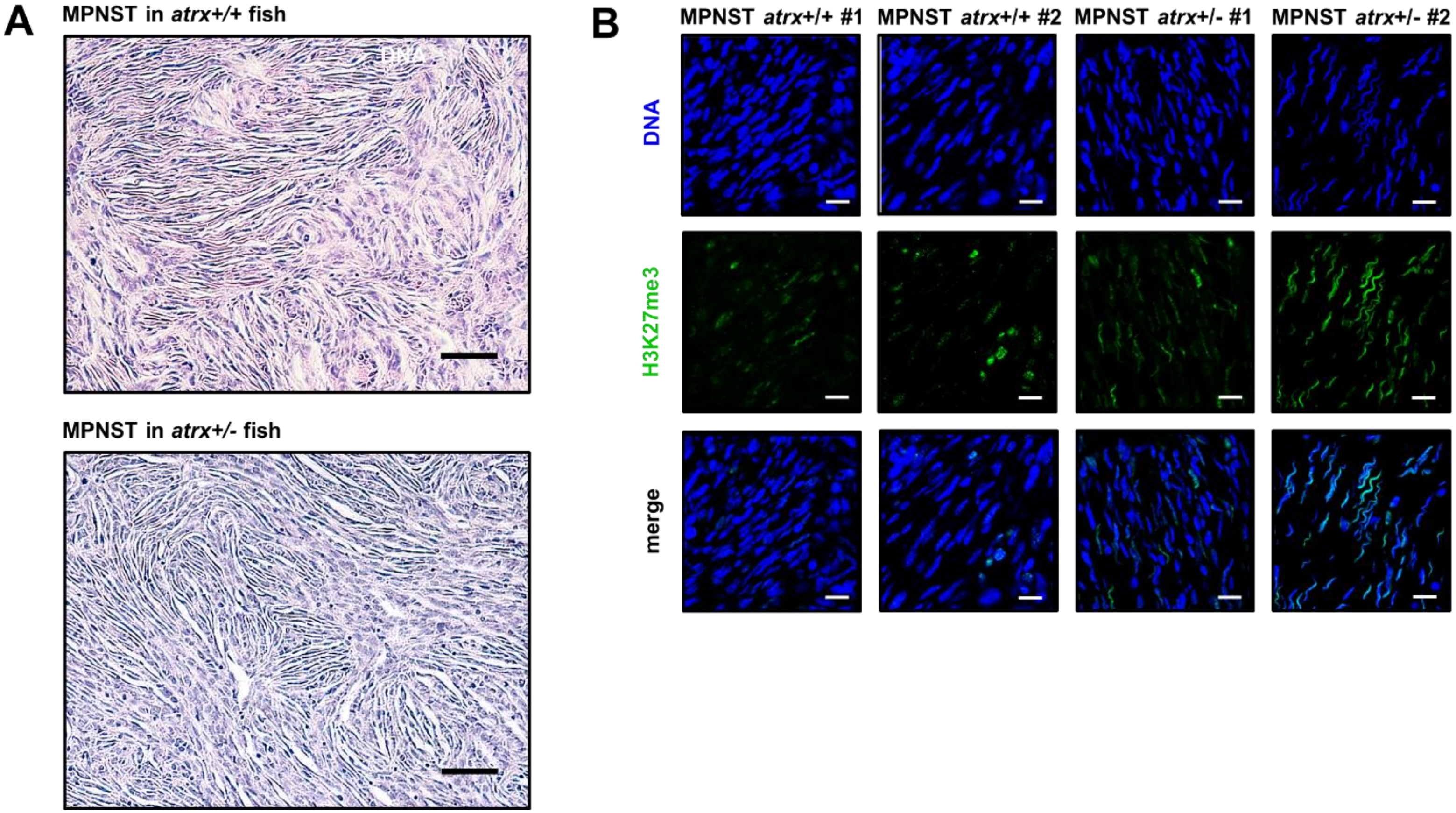

S3 Fig: MPNST biology in atrx+/+ and atrx+/- siblings in p53/nf1-deficient background. (A) HE-staining of p53/nf1 atrx+/+ and atrx+/- MPNSTs reveals no differences in histology; scale bars: 50µm. **(B)** Indirect immunofluorescence staining of two MPNSTs each of the p53/nf1 atrx+/+ and atrx+/- cohorts both show detectable tri-methylation of histone 3, lysine 27 (H3K27me3, green); scale bars: 10µm.
