# Supplementary material for: Loss of atrx cooperates with p53-deficiency to promote the development of sarcomas and other malignancies
Source: PLoS Genet. 2019 Apr 10;15(4):e1008039. doi: 10.1371/journal.pgen.1008039 (PMC6476535; doi:10.1371/journal.pgen.1008039)
Supplement: S4 Fig — Significantly up- or downregulated genes (p<0.025) between p53-/-, nf1b-/-, nf1a+/-, atrx+/+ and p53-/-, nf1b-/-, nf1a+/-, atrx+/- samples (n = 3). The heat map is row normalized with blue representing minimum expression and red representing maximum expression. (PDF) [file pgen.1008039.s004.pdf]

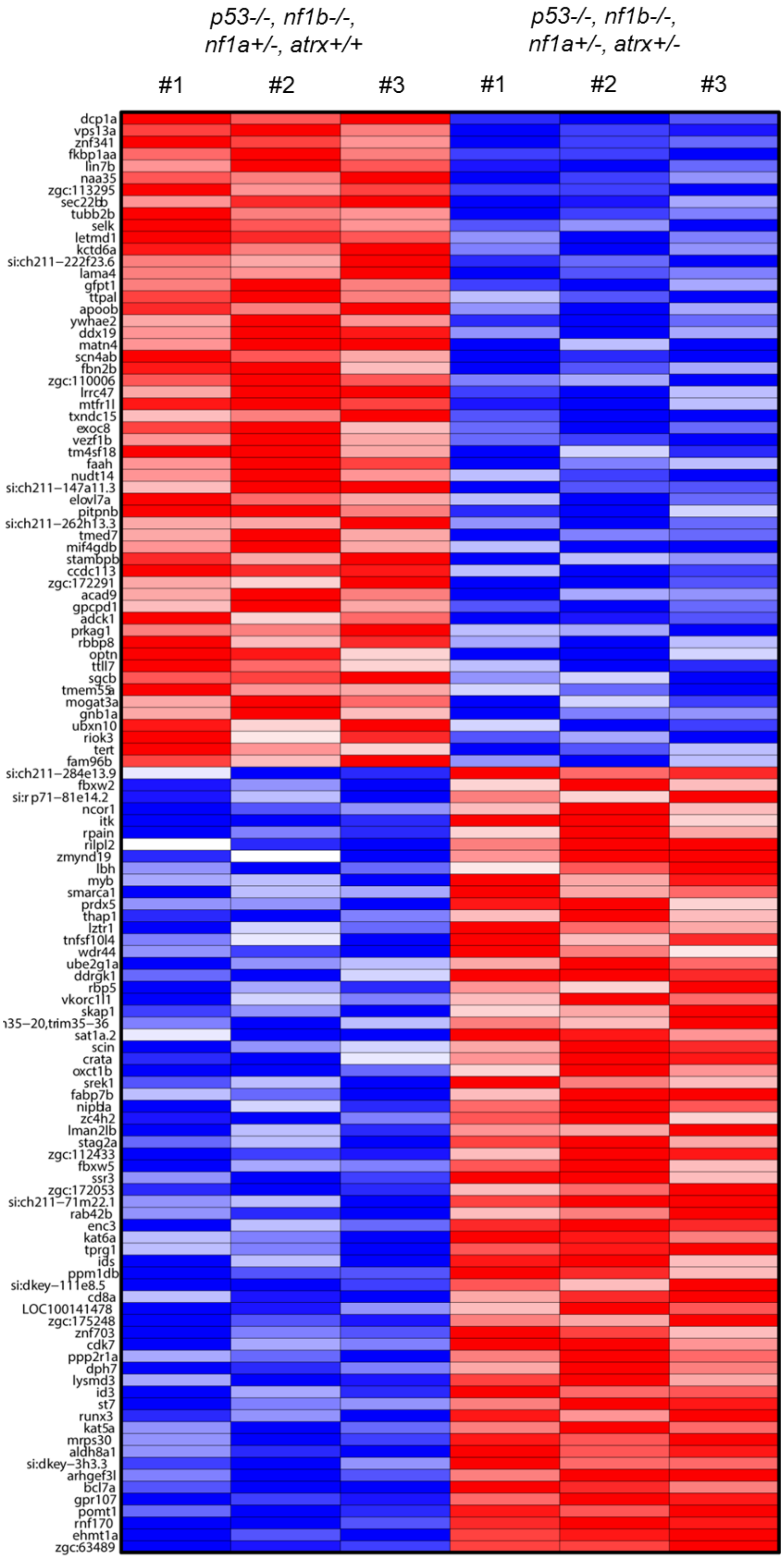

S4 Fig: Head map of RNA-Seq results. Significantly up- or downregulated genes ($p<0.025$) between p53-/-, nf1b-/-, nf1a+/-, atrx+/+ and p53-/-, nf1b-/-, nf1a+/-, atrx+/- samples (n=3). The heat map is row normalized with blue representing minimum expression and red representing maximum expression.
