# Supplementary material for: Loss of atrx cooperates with p53-deficiency to promote the development of sarcomas and other malignancies
Source: PLoS Genet. 2019 Apr 10;15(4):e1008039. doi: 10.1371/journal.pgen.1008039 (PMC6476535; doi:10.1371/journal.pgen.1008039)
Supplement: S5 Fig — RNA-Seq analysis revealed significantly increased expression of PRC2-related gene sets, NOTCH1 and JAK/STAT signaling targets and markers of epithelial differentiation; group 1: p53-/-, nf1b-/-, nf1a+/-, atrx+/+ (control); group 2: p53-/-, nf1b-/-, nf1a+/-, atrx+/-. (PDF) [file pgen.1008039.s005.pdf]

PRC2-related gene sets

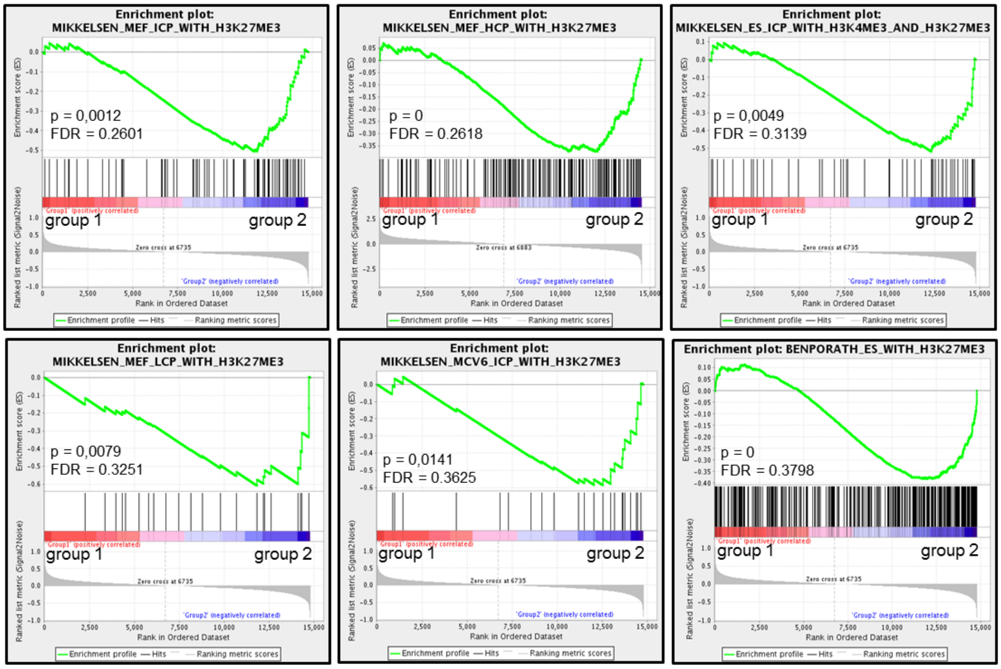

Signaling pathways

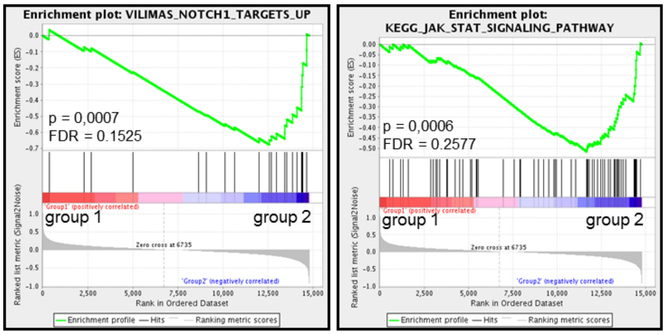

Epithelial differentiation

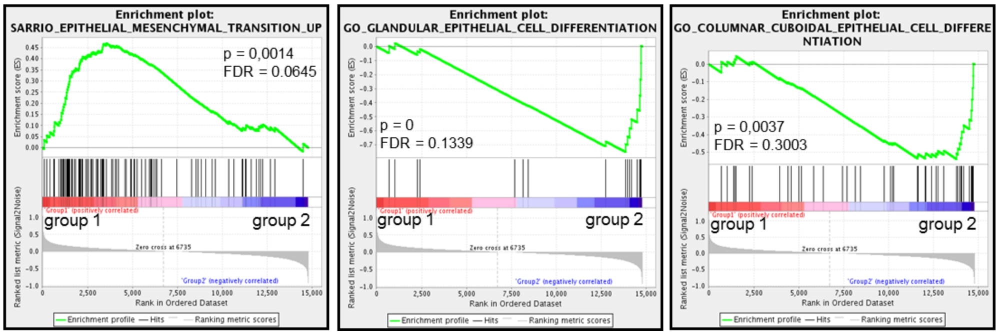

S5 Fig: Gene set enrichment analysis. RNA-Seq analysis revealed significantly increased expression of PRC2-related gene sets, NOTCH1 and JAK/STAT signaling targets and markers of epithelial differentiation; group 1: p53-/-, nf1b-/-, nf1a+/-, atrx+/+ (control); group 2: p53-/-, nf1b-/-, nf1a+/-, atrx+/-.
