# Supplementary material for: Loss of atrx cooperates with p53-deficiency to promote the development of sarcomas and other malignancies
Source: PLoS Genet. 2019 Apr 10;15(4):e1008039. doi: 10.1371/journal.pgen.1008039 (PMC6476535; doi:10.1371/journal.pgen.1008039)
Supplement: S1 Table — (PDF) [file pgen.1008039.s006.pdf]

S1 Table: Germline *atrx* mutations in F1 in *p53/nf1*-deficient background

F0 fish #	F1 fish #	Mutation	Germline sequence	
Wildtype			TTCCGAGGT----CCTGA----GTT-CC---G----TAA-----------------CAAG----GGA	
1	1	3del	TTCCGAGGT----CCTGA----GTT-CC---G----TAA-----------------CAAG----GGA	
	2	3del	TTCCGAGGT----CCTGA----GTT-CC---G----TAA-----------------CAAG----GGA	
2	1	3del	TTCCGAGGT----CCTGA----GTT-CC---------AT------------------AAG----GGA	
	2	7del	TTCCGAGGT----CCTGA----GTT-CC-------------------------------G----GGA	
	3	2in	TTCCGAGGT----CCTGA----GTT-CC--------TGAGTT--------------CAAG----GGA	Line 1
	4	2in	TTCCGAGGT----CCTGA----GTT-CC--------TGAGTT--------------CAAG----GGA	
	5	1in	TTCCGAGGT----CCTGA----GTT-CC--------TTT------------------AAG----GGA	
	6	7del	TTCCGAGGT----CCTGA----GTT-CC-------------------------------G----GGA	
3	1	3del	TTCCGAGGT----CCTGA----GTT-CC---G----T--------------------AAG----GGA	
	2	22in	ACTGGTGGTGAAGCCAGAACCAGTTGCTAATGAAACTAAAGATGC-----------CAAG----GGA	Line 2
	3	3del	TTCCGAGGT----CCTGA----GTT-CC---G----T--------------------AAG----GGA	
4	1	7in	TTCCGAGGT----CCTGA----GTT-CC---G-----AGGTCCTG------------A-GTTCCGGA	
	2	17in	TTCCGAGGT----CCTGA----GTT-CC---G----TAAGGGTTCCGTAAGTTTGCCAAG----GGA	
	3	7in	TTCCGAGGT----CCTGA----GTT-CC---G-----AGGTCCTG------------A-GTTCCGGA	
5	1	3del	TTCCGAGGT----CCTGA-----TTTCC---G----TAA--------------------G----GGA	
6	1	3del	TTCCGAGGT----CCTGA----GTT-CC---G----TAA--------------------G----GGA	
	2	3del	TTCCGAGGT----CCTGA----GTT-CC---G----T--------------------AAG----GGA	
	3	3del	TTCCGAGGT----CCTGA----GTT-CC---G----TAA--------------------G----GGA	
7	1	3sub	TTCCGAGGT----CCTGA----GTT-CC---G----TAAGG----------------A-G----GCA	
	2	12del	TTCCGAGGT----CCTGA----G--------G---------------------------G------A	
8	1	3del	TTCCGAGGT----CCTGA----GTT-CC---G----T--------------------AAG----GGA	
	2	3del	TTCCGAGGT----CCTGA----GTT-CC---G----T--------------------AAG----GGA	
9	1	5del	TTCCGAGGT----CCTGA----GTT-C------------------------------CAAG----GGA	
	2	3del	TTCCGAGGT----CCTGA----GTT-CC---G----T--------------------AAG----GGA	
10	1	12in	TTCCGAGGT----CCTGA----GTT-CC---G----T------------ACTACAA------GGTAGTTCAAG	
n=10	n=25	n=13		

del = deletion; in = insertion; sub = substitution
