# Supplementary material for: Loss of atrx cooperates with p53-deficiency to promote the development of sarcomas and other malignancies
Source: PLoS Genet. 2019 Apr 10;15(4):e1008039. doi: 10.1371/journal.pgen.1008039 (PMC6476535; doi:10.1371/journal.pgen.1008039)
Supplement: S3 Table — (PDF) [file pgen.1008039.s008.pdf]

S3 Table: Analysis of *ATRX* mutation frequency in human malignancies in AACR Genie Database

Cancer Type	# samples analyzed	# *ATRX* mutant	Frequency %
Uterine Sarcoma	199	39	19.60
Glioma	2168	363	16.74
Blastic Plasmacytoid Dendritic Cell Neoplasm	6	1	16.67
Myeloproliferative Neoplasm	182	27	14.84
Pheochromocytoma	17	2	11.76
Pineal Tumor	10	1	10.00
Adrenocortical Carcinoma	98	9	9.18
Small Cell Lung Cancer	236	21	8.90
Skin Cancer, Non-Melanoma	344	30	8.72
Miscellaneous Neuroepithelial Tumor	38	3	7.89
Leukemia	1150	87	7.57
Anal Cancer	95	7	7.37
Endometrial Cancer	1076	77	7.16
Embryonal Tumor	214	14	6.54
Soft Tissue Sarcoma	1200	77	6.42
Penile Cancer	18	1	5.56
Bladder Cancer	1067	58	5.44
Myelodysplasia	268	14	5.22
Gastrointestinal Neuroendocrine Tumor	199	10	5.03
Melanoma	1868	93	4.98
Non-Small Cell Lung Cancer	6346	244	3.84
Bone Cancer	354	13	3.67
Vaginal Cancer	56	2	3.57
Pancreatic Cancer	1298	45	3.47
Nerve Sheath Tumor	31	1	3.23
Esophagogastric Cancer	1072	33	3.08
Cervical Cancer	271	8	2.95
Small Bowel Cancer	136	4	2.94
Breast Sarcoma	34	1	2.94
Colorectal Cancer	4335	124	2.86
Ovarian Cancer	1473	39	2.65
Sellar Tumor	153	4	2.61
Appendiceal Cancer	230	6	2.61
Multiple Myeloma	39	1	2.56
Salivary Gland Cancer	381	9	2.36
Head and Neck Cancer	819	19	2.32
Breast Cancer	4506	93	2.06
Non-Hodgkin Lymphoma	475	9	1.89
CNS Cancer	270	5	1.85

Thyroid Cancer	681	12	1.76
Renal Cell Carcinoma	967	13	1.34
Miscellaneous Brain Tumor	87	1	1.15
Hepatobiliary Cancer	804	9	1.12
Mesothelioma	333	3	0.90
Prostate Cancer	1488	9	0.60
Gastrointestinal Stromal Tumor	405	2	0.49
Germ Cell Tumor	488	1	0.20
